# Influence of diabetes on response to ultrasound guided hydrodistension treatment of adhesive capsulitis: a retrospective study

**DOI:** 10.1186/s12902-022-01144-x

**Published:** 2022-09-12

**Authors:** Sofia Dimitri-Pinheiro, Beatriz Serpa Pinto, Madalena Pimenta, João Sérgio Neves, Davide Carvalho

**Affiliations:** 1 Radiology Department, Portuguese Institute of Oncology of Porto – Francisco Gentil EPE, Porto, Portugal; 2 Biomedicine Department, Unit of Biochemistry, Faculty of Medicine, University of Porto, Porto, Portugal; 3 Faculty of Medicine, University of Porto, Alameda Professor Hernâni Monteiro, 4200-319 Porto, Portugal; 4 CUF Hospital, Porto, Portugal; 5 Endocrinology Department, São João University Hospital Centre, Porto, Portugal; 6 Department of Surgery and Physiology, Cardiovascular Research Unit, Faculty of Medicine, University of Porto, Porto, Portugal; 7 I3S - Institute for Innovation and Health Research, University of Porto, Porto, Portugal

**Keywords:** Diabetes mellitus, Adhesive capsulitis, Shoulder, Musculoskeletal disorders, Diabetes complications, Ultrasound guided hydrodistension

## Abstract

**Background:**

Diabetes is associated with microvascular and macrovascular complications. Although it is less recognized, diabetes also has an important role in the development of musculoskeletal disorders. Our objective was to evaluate the effect of type 2 diabetes (T2D) on the severity of adhesive capsulitis of the shoulder (AC) and on the efficacy of ultrasound guided hydrodistension treatment.

**Methods:**

We conducted a retrospective longitudinal observational study, of patients with AC who underwent ultrasound guided hydrodistension at our Centre. Severity was measured with DASH (Disabilities of Arm, Shoulder and Hand) score and pain was evaluated with a score between 0 and 10. The association of T2D with baseline characteristics of AC, and with outcomes at 6–12 months was analyzed using linear and logistic regression models.

**Results:**

We evaluated 120 ultrasound guided hydrodistension treatments of AC, 85 in patients without diabetes and 35 in patients with T2D. Patients with diabetes had a higher prevalence of dyslipidemia, hypertension and higher HbA1c values. The average duration of diabetes was 4.8 years (2.0, 7.9). The baseline characteristics of AC were not significantly different between patients with and without diabetes. Patients with T2D relapsed more frequently and required more reinterventions than patients without diabetes (20.0% vs 4.7%, *p* = 0.008), had higher post-intervention pain scale values [4.0 (0.0–5.0) vs 0.0 (0.0–5.0), *p* = 0.022] and higher post-intervention DASH score [0.8 (0.0–1.8) vs 0.0 (0.0–0.8), *p* = 0.038].

**Conclusion:**

Although baseline characteristics of AC in patients with diabetes were similar to those without diabetes, patients with diabetes had a worse response to treatment, more frequent relapses and a greater need for new interventions.

## Background

Diabetes affects every organ and system and is associated with several microvascular and macrovascular complications [1]. Although it is less recognized, diabetes also has an important role in the development of several musculoskeletal disorders, such as the adhesive capsulitis of the shoulder (AC) [2, 3].

AC or commonly called “frozen shoulder” is characterized by contraction of the glenohumeral capsule and adherence to the head of the humerus. Clinically, is characterized by diffuse pain of the shoulder, with an insidious onset and evolution over a few weeks. There is usually nocturnal worsening and can lead to a limitation of the active and passive range of motion of the joint, which ultimately can progress to a complete blockage of mobility [4]. AC is usually idiopathic/primary. However, it can also be secondary to various illnesses including diabetes [5, 6]. Studies suggest that patients with diabetes have a higher incidence of AC with more severe disease (with more pain and functional impairment) [3, 7, 8]. The prevalence of AC in patients with diabetes is between 11 and 30%, whereas in patients without diabetes it is 2–10% [3]. *Zreik* et al described that patients with type 2 diabetes (T2D) were 5 times more likely than controls to have AC [9]. The strong association between diabetes and the development of AC has led several authors to advocate screening for diabetes in patients with AC [10–12].

In most cases, AC is self-limited, with mild symptoms and can be effectively managed using analgesics, physiotherapy and intra-articular injections of corticosteroids. However, in 20 to 50% of patients, the symptoms may persist and, in such cases, treatment with capsule hydrodistension should be considered [7]. Ultrasound guided hydrodistension consists of the injection, under ultrasound guidance, of a combination of corticosteroids and saline solution inside the glenohumeral capsule. There is physical distension and release of the capsule by the injected liquid [7].

Our objective was to evaluate how diabetes influences the severity of AC, in terms of pain and functional impairment, as well as how diabetes influences response to ultrasound guided hydrodistension treatment. We also evaluated whether this intervention is metabolically safe for patients with diabetes.

## Materials and methods

### Study design and participants

We performed a retrospective longitudinal observational study at Centro Hospitalar Universitário de São João (CHUSJ) in Porto, Portugal, to evaluate how diabetes influences the severity of AC, as well as response to ultrasound-guided hydrodistension treatment. Patients with clinical and ultrasound diagnosis of AC, who underwent ultrasound-guided hydrodistension between 2016 and 2018 and who were 18 years or older at the last time evaluated were included. Clinical diagnosis of AC was defined as shoulder pain for at least 1 month, inability to lie on the affected shoulder and restricted active and passive shoulder joint mobility in at least three planes on physical examination. Ultrasound diagnosis of AC was established in patients with clinical diagnosis of AC and a thickened coracohumeral ligament, thickened inferior capsule, hipoechogenicity and increased Doppler signal in the rotator cuff interval. The research project was approved by the Ethics Committee of CHUSJ / Faculty of Medicine, Universidade do Porto.

Data from 2 different moments was collected from patient’s electronic clinical records: before ultrasound guided hydrodistension (up to 6 months before) and at 6–12 months after the intervention. To obtain data regarding the pain scale and DASH (Disabilities of Arm, Shoulder and Hand) score before and after intervention, patients were surveyed by telephone. A 0–10 pain scale (0 equals no pain and 10 the maximum pain intensity) and a modified DASH score were used (0 correspond to the minimum shoulder disability and 100 correspond to the maximum shoulder disability). The same participant could be included multiple times if a reintervention was performed during the study period.

### Clinical characteristics and study outcomes

The following clinical parameters were collected: sex, age, diagnosis of diabetes, age at diagnosis of diabetes, treatment with antidiabetic drugs, hypertension, dyslipidemia, body mass index (BMI), current smoking, fasting blood glucose, HbA1c, lipid profile (total cholesterol, HDL, LDL, triglycerides), pain scale (0 to 10), DASH score, which shoulder was intervened (right or left) and whether it was bilateral or unilateral.

The main outcomes of the present study were: pain scale after the intervention, DASH score after the intervention, variation of the pain scale, variation of the DASH score, and relapse and need for a new intervention.

Additionally, we considered metabolic outcomes in patients with diabetes including HbA1c, fasting blood glucose, total cholesterol, LDL cholesterol, HDL cholesterol and triglycerides. We compared the values of these parameters before the intervention and 3 to 6 months after the intervention.

### Statistical analysis

The comparison of baseline characteristics between the two groups was performed using Student’s t-test or Wilcoxon rank-sum test for normal and non-normal continuous variables respectively, and the chi-square test for categorical variables. For the baseline characteristics only the first intervention was considered in patients with multiple interventions. The association of predictors with study outcomes was evaluated using linear regression (for continuous outcomes) and logistic regression (for dichotomic outcomes). Continuous variables are described as mean ± standard deviation or median (25th percentile - 75th percentile) and categorical variables as proportions (percentages). A bilateral *p* value of < 0.05 was considered statistically significant. Analyzes were performed with Stata (version 14.2).

## Results

During the study period, 120 ultrasound guided hydrodistension procedures were performed. Of these, 109 patients were submitted to only one intervention and 11 were submitted to multiple interventions. The baseline characteristics of the 109 patients (80 without diabetes and 29 with diabetes) are shown in Table 1. Regarding patients without diabetes, 85.0% were women, the average age was 57.9 ± 11.2 years, 53.8% had dyslipidemia, 33.8% had hypertension, 8.9% smoked, the mean BMI was 27.0 ± 4.2 kg/m^2^, the mean HbA1c was 5.9 ± 0.5% the and mean fasting plasma glucose was 89.4 ± 13.9 mg/dL. In what concerns to patients with diabetes, 75.9% were women, the average age was 61.9 ± 8.9 years, 75.9% had dyslipidemia, 82.8% had hypertension, 10.3% smoked, the mean BMI was 28.2 ± 4.5 kg/m^2^, the mean HbA1c was 7.0 ± 1.0% and the mean fasting plasma glucose 148.5 ± 101.1 mg/dL. There were no significant differences regarding sex, age, smoking and BMI. Patients with diabetes had a higher prevalence of dyslipidemia and hypertension.

**Table 1:** Baseline characteristics

	Patients without Diabetes	Patients with T2D	*P* value
	(*N* = 80)	(*N* = 29)	
Female sex, n (%)	68 (85.0%)	22 (75.9%)	0.27
Age, years	57.9 ± 11.2	61.9 ± 8.9	0.08
Dyslipidemia, n (%)	43 (53.8%)	22 (75.9%)	0.038
Hypertension, n (%)	27 (33.8%)	24 (82.8%)	0.001
Current smoking, n (%)	7 (8.9%)	3 (10.3%)	0.81
Body Mass Index, kg/m^2^	27.0 ± 4.2	28.2 ± 4.5	0.42
HbA1c, %	5.9 ± 0.5	7.0 ± 1.0	0.031
Fasting plasma glucose, mg/dL	89.4 ± 13.9	148.5 ± 101.1	0.001

Table 2 presents data regarding diabetes duration and diabetes treatment in patients with diabetes. The average duration of diabetes was 4.8 years (2.0–7.9). Most patients were treated with metformin (85.7%). There were no patients treated with GLP1 (Glucagon-like peptide-1) receptor agonists or SGLT2 (Sodium-glucose cotransporter 2) inhibitors.

**Table 2:** Diabetes duration and diabetes treatment

Duration of diabetes, years	4.8 (2.0, 7.9)
Metformin, n (%)	30 (85.7%)
DPP4 inhibitors, n (%)	8 (22.9%)
Pioglitazone, n (%)	1 (2.9%)
Sulfonylureas, n (%)	1 (2.9%)
Insulin therapy, n (%)	4 (11.4%)

Diabetes duration and diabetes drugs

*DPP4 inhinitors* inhibitors of dipeptidyl peptidase 4

Table 3 shows the characteristics of AC patients before ultrasound guided hydrodistension procedures. There were no differences in which shoulder was intervened (right or left) and whether it was bilateral or unilateral. The DASH score and the pain scale before intervention were similar in both groups.

**Table 3:** Baseline characteristics of AC of the Shoulder

	Patients without diabetes (*N* = 85)	Patients with T2D (*N* = 35)	*P* value
Right Shoulder, n (%)	37 (43.5%)	19 (54.3%)	0.28
Bilateral, n (%)	6 (7.1%)	4 (11.4%)	0.43
DASH score	94.2 (69.2, 94.2)	94.2 (69.2, 94.2)	0.70
Pain Scale	10.0 (8.0, 10.0)	10.0 (8.0, 10.0)	0.88

*DASH* disabilities of arm, shoulder and hand

Table 4 shows the outcomes of ultrasound guided hydrodistension treatment of AC. Regarding relapse and reintervention, 4.7% of patients without diabetes relapsed and required a new intervention, and 20.0% of patients with diabetes relapsed and needed a new intervention. Thus, relapse and need for new intervention was more frequent in patients with diabetes (*p* = 0.008).

**Table 4:** Outcomes of ultrasound guided hydrodistension treatment of AC of the Shoulder

	Patients without diabetes (*N* = 85)	Patients with T2D (*N* = 35)	*P* value
Relapse and need for new intervention, n (%)	4 (4.7%)	7 (20.0%)	0.008
Pain scale after intervention	0.0 (0.0, 5.0)	4.0 (0.0, 5.0)	0.022
Variation of pain scale	−8.0 (−10.0, −5.0)	−5.0 (− 8.0, − 4.0)	0.036
DASH score after intervention	0.0 (0.0, 0.8)	0.8 (0.0, 1.8)	0.038
Variation of DASH score	− 93.4 (−94.2, − 69.2)	− 92.4 (− 93.4, − 69.2)	0.10

*DASH* disabilities of arm, shoulder and hand

The median pain scale after the intervention in patients with diabetes was 4 and in patients without diabetes was 0. The median of DASH score after the intervention in patients without diabetes was 0.0 and in patients with T2D it was 0.8. Thus, after the intervention, patients with diabetes had higher pain scale (*p* = 0.022) and DASH score (*p* = 0.038) comparing with patients without diabetes.

There was a significantly smaller decrease in the pain scale in patients with diabetes comparing with patients without diabetes [− 5.0 (− 8.0, − 4.0) vs − 8.0 (− 10.0, − 5.0), *p* = 0.36]. The difference in the variation of DASH score was not statistically significant between patients with and without diabetes*.*

Table 5 shows the metabolic outcomes of patients with diabetes before and 3–6 months after the intervention. There were no significant differences regarding weight, HbA1c, fasting plasma glucose, total cholesterol, LDL cholesterol, HDL cholesterol and triglycerides, before and after the intervention.

**Table 5:** Metabolic outcomes in patients with diabetes

	Before intervention	3–6 months after intervention	*P* Value
Weight, kg	67.6 ± 10.6	68.3 ± 11.6	0.30
HbA1c, %	6.7 ± 1.1	6.6 ± 1.2	0.46
Fasting blood glucose, mg/dL	102.0 ± 34.2	106.0 ± 25.3	0.34
Total cholesterol, mg/dL	193.3 ± 25.4	185.3 ± 29.2	0.14
LDL cholesterol, mg/dL	108.0 ± 24,0	105.2 ± 23.7	0.58
HDL cholesterol, mg/dL	61.7 ± 12.5	59.7 ± 8.9	0.30
Triglycerides, mg/dL	97.5 ± 45.6	93.6 ± 36.0	0.75

## Discussion and conclusions

The aim of this study was to understand the influence of diabetes on AC and on the response to treatment with ultrasound guided hydrodistension. There are five key findings of the present research. First, there were no significant differences between patients with and without diabetes, regarding the predominance of the right shoulder, the disease being bilateral, the DASH score before the intervention and the pain scale before the intervention. Second, relapse and need for new intervention was higher in patients with diabetes. Third, after the intervention, patients with diabetes had higher pain scale and DASH score. Fourth, there was a smaller reduction in pain scale in patients with diabetes comparing with patients without diabetes, and a trend for a smaller decrease in the DASH score. Fifth, there were no significant differences regarding weight, HbA1c, fasting glucose and lipid profile, before and after the intervention in patients with diabetes.

Our results are consistent with the previous literature that suggests that patients with diabetes have worse responses to treatment [13, 14]. Other studies have suggested that patients with diabetes have a higher incidence of AC, however our study was not designed to compare the incidence of AC between groups [2, 3, 15]. Whether patients with diabetes have more severe disease is unclear. In our study, there were no significant differences between patients with and without diabetes regarding DASH score and pain scale before the intervention. This is in accordance with the study by *Uddin* et al that found no differences in level of pain and disability level between AC in patients with and without diabetes [16]. On the other hand, most studies have suggested that AC in patients with diabetes is associated with more pain and functional impairment [3, 7, 8].

Diabetes is frequently associated with other metabolic diseases including dyslipidemia, as observed in our study. Whether dyslipidemia also contributes to the development of AC and with the response to treatment is still uncertain. A previous study about this found that hyperlipidemia in patients with diabetes was an independent risk factor for AC [17]. Some studies observed an association of inflammatory lipoproteins, particularly higher levels of LDL cholesterol and higher levels of non-HDL cholesterol, with AC in patients with diabetes [12, 18].

The lower response rate to treatment of AC among patients with diabetes has also been described with other interventions. The conservative treatment of AC (with analgesics and/or physiotherapy) has lower response rates in those with diabetes [7]. The same pattern is observed with surgical treatment of AC. Boutefnouchet et al. observed that, in comparison with patients without diabetes, patients with diabetes had more residual pain, reduced motion and lower function after surgery [19]. Yanlei et al. found that patients with diabetes had poorer improvement in internal rotation and forward flexion postoperatively [20]. Pollock et al. and Su et al. also described worse functional outcomes among those with diabetes [21, 22]. Our study reinforces the notion that the response to therapeutic interventions in AC of the shoulder is worse in patients with diabetes.

Several mechanisms may explain the association of AC with diabetes. Chronic hyperglycemia, increased visceral adiposity and chronic inflammation of diabetes may have central roles in this association [23]. Chronic hyperglycemia stimulates the development of cross-links between collagen molecules. Due to these cross-links, the breakdown of collagen molecules is impaired, eventually accumulating in cartilage, ligaments, tendon sheaths and in the tendons. Patients with diabetes have an increase in visceral adipocytes, which secrete cytokines, such as tumor necrosis factor alpha (TNF-a) and interleukin-6 (IL-6), resulting in the overproduction of other pro-inflammatory cytokines [23]. This pro-inflammatory environment will exacerbate inflammation and insulin resistance. In turn, fatty acids secreted by adipocytes promote neutrophil survival and decrease the removal of apoptotic cells by phagocytic cells, increasing collagen deposition in the glenohumeral capsule, giving rise to a stiff and painful shoulder [23]. Finally, chronic inflammation results in excessive accumulation of collagen and other extracellular matrix components, with destruction of the normal tissue architecture in the joints. Thus, these 3 factors perpetuate the disease [23–27].

As it is known, corticosteroids have several potential systemic adverse effects and most studies have not evaluated if ultrasound guided hydrodistension treatment (with injection of a combination of corticosteroids and saline solution inside the glenohumeral capsule) is associated with fluctuations of blood glucose levels or lipid profile. In our study, we found no significant differences regarding weight, HbA1c, fasting plasma glucose and lipid profile, before and after the intervention. Therefore, our results suggest that the intervention is safe from a metabolic point of view, not interfering with weight, glycemic and lipid profile.

There are two relevant limitations of our study. The first limitation concerns the retrospective design of our study, which limits the collection of data. The second limitation is that the study was carried out in a single center, which may decrease the generalizability of our findings. Therefore, more studies are required to better understand the influence of diabetes on AC. Furthermore, only a small proportion of the patients included in our analysis were men. Although AC of the shoulder is known to be more common in women, the generalizability of our results to the male population must be performed with caution. Regarding the strengths of our study, we included patients with detailed data regarding AC treatment and diabetes-related variables with the comparison of baseline characteristics of AC, as well as the effects of ultrasound guided hydrodistension on pain and functional disability. The evaluation of metabolic effects of the intervention among those with diabetes is also an important contribution of our study to the understanding of the safety of ultrasound guided hydrodistension.

More studies are needed to better understand the extent of the influence of diabetes on AC and on the response to treatment with ultrasound guided hydrodistension. In terms of future research, it would be relevant to evaluate the influence of strict control of glucose levels on treatment response. It could be also of interest to determine how different doses of corticosteroid affect the procedure efficacy. Although many studies assess the role of diabetes on the incidence of AC, few investigate the role of diabetes on the response to treatment with ultrasound guided hydrodistension. With our study demonstrating a clear role for diabetes on treatment response, we hope that our research stimulates further investigation in this area.

In conclusion, although the baseline characteristics of AC in patients with diabetes were similar to those without diabetes, patients with diabetes had a worse response to treatment, more frequent relapses and a greater need for new interventions. A greater awareness of the association of diabetes with musculoskeletal disorders, particularly with AC, is needed, to ensure an earlier recognition and treatment to improve its prognosis.

## Data Availability

The datasets generated and analyzed during the current study are not publicly available due to containing information that could compromise the privacy of the research participants, but are available from the corresponding author on reasonable request.
